# SARS-CoV-2 Delta Variant among Asiatic Lions, India

**DOI:** 10.3201/eid2710.211500

**Published:** 2021-10

**Authors:** Anamika Mishra, Naveen Kumar, Sandeep Bhatia, Ashutosh Aasdev, Sridhar Kanniappan, Abelraj Thaya Sekhar, Aparna Gopinadhan, Ramu Silambarasan, Chirukandoth Sreekumar, Chandan Kumar Dubey, Meghna Tripathi, Ashwin Ashok Raut, Vijendra Pal Singh

**Affiliations:** ICAR–National Institute of High Security Animal Disease, Bhopal, India (A. Mishra, N. Kumar, S. Bhatia, A. Aasdev, C.K. Dubey, M. Tripathi, A.A. Raut, V.P. Singh);; Arignar Anna Zoological Park, Chennai, India (S. Kanniappan, A.T. Sekhar, A. Gopinadhan, R. Silambarasan);; Madras Veterinary College, Chennai (C. Sreekumar);; All India Institute of Medical Sciences, Bhopal (A.A. Raut)

**Keywords:** respiratory infections, severe acute respiratory syndrome coronavirus 2, SARS-CoV-2, SARS, COVID-19, coronavirus disease, zoonoses, viruses, coronavirus, Delta variant, Asiatic lions, India

## Abstract

In May 2021, severe acute respiratory syndrome coronavirus 2 (SARS-CoV-2) was detected in Asiatic lions in a zoological park in India. Sequence and phylogenetic analyses showed the SARS-CoV-2 strains were the B.1.617.2 (Delta) variant. To reduce transmission of variants of concern, surveillance of SARS-CoV-2 in wild animal populations should be increased.

Severe acute respiratory syndrome coronavirus 2 (SARS-CoV-2), in natural conditions, has shown a broad host susceptibility range (*1*). Identifying susceptible animal species, reservoirs, and cross-species transmission is a global scientific and public health concern. We found evidence of natural SARS-CoV-2 infection in Asiatic lions (*Panthera leo persica*) caused by the lineage B.1.617.2 (Delta) variant (World Health Organization nomenclature). We provide coronavirus disease (COVID-19) case information and detailed genomic characterization.

Arignar Anna Zoological Park in Chennai, India, houses 13 Asiatic lions, 9 in a lion safari and 2 each in separate moat enclosures. Beginning May 21, 2021, four of the safari lions started showing signs of loss of appetite, nasal discharge, and occasional coughing. Nasal swab, rectal swab, and fecal samples were collected from 11 lions during May 24–29, 2021, and sent to the Indian Council of Agricultural Research–National Institute of High Security Animal Diseases (Bhopal, India) for molecular investigations (Appendix Table 1). 

We used the VIRALDTECT II Multiplex Real Time PCR Kit for COVID-19 (Genes2Me, https://genes2me.com) to confirm SARS-CoV-2 in 9/11 lions. The other 2 lions were sampled on June 19, 2021, and tested negative for SARS-CoV-2. We also used a World Organisation for Animal Health–recommended reverse transcription PCR (RT-PCR) method to test for canine distemper virus on samples from all 13 lions; all tested negative (*2*). Two of the infected lions died of COVID-19, one on June 3 and the other on June 16, 2021. 

After we confirmed SARS-CoV-2 infection, we performed whole-genome sequencing directly from nasal swab specimens of 4 lions that initially showed symptoms by using the MinION sequencing platform (Oxford Nanopore Technologies, https://nanoporetech.com) (Appendix). We deposited sequences in GenBank (accession nos. MZ363851–4) and GISAID (https://www.gisaid.org; accession nos. EPI_ISL_2821077–80).

To elucidate the temporal dynamics of SARS-CoV-2 among the lions, we downloaded 310 complete SARS-CoV-2 genomes from GISAID (*3*) that had high coverage and were sequenced from the Tamil Nadu state of India, where the zoological park is located, during January 1–June 11, 2021. To generate a set of representative sequences, we used a UCLUST algorithm (*4*) to select sequences that clustered at the 99.9% identity threshold. We used MAFFT version 7.475 (*5*) to align representative SARS-CoV-2 sequences from GISAID with sequences from the lions; then we constructed a phylogenetic tree by using the general time reversible plus gamma model in RAxML version 8.2.12 (*6*) (Figure).

**Figure:**
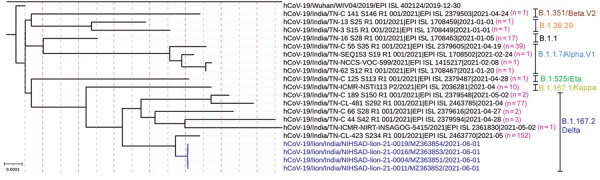
Complete genome phylogenetic analysis of severe acute respiratory syndrome coronavirus 2 (SARS-CoV-2) detected in Asiatic lions (*Panthera leo persica*), India (blue text), and representative sequences of different clusters generated at 99.9% identity threshold from the available SARS-COV-2 sequences from Tamil Nadu, India, in the GISAID. The maximum-likelihood tree was rooted to Wuhan-Hu-1 reference sequence (GISAID accession no. EPI_ISL_402124). GenBank accession numbers are provided for the sequences from this study. Pink numbers in parentheses indicate the number of SARS-CoV-2 genome sequences clustered at 99.9% identity threshold. Other text colors represent SARS-CoV-2 variants. Scale bar indicates nucleotide substitutions per site.

The amino acid substitutions and deletions in the spike protein of SARS-CoV-2 in lions typically matched with the SARS-CoV-2 Delta variant (Appendix Table 2). We noted amino acid changes in the N terminal domain (NTD), including T19R, G142D, E156del, F157del, R158G; in the receptor binding motif (RBM), including L452R and T478K; and in D614G of subdomain 2. We also noted a substitution close to S1/S2 protease cleavage site at P681R and heptad repeat 1 at D950N (Appendix Figures 1, 2). In addition, the lion sequences had the K77T substitution in the NTD, which has been detected in SARS-CoV-2 genomes from 24 countries. In India, frequency of the K77T substitution generally is low (0.44%) but occurred in 27.42% (65/237) of sequences in the B.1.167.2 lineage collected in Tamil Nadu state (Appendix Table 2).

The changes in the spike protein, including E156del, F157del, and R158G, of lion sequences were not found in human SARS-CoV-2 sequences from the same geographic area, nor were changes in nonstructural protein 3 (NS3) V88I, possibly because SARS-CoV-2 sequencing is limited in the region. Furthermore, these changes in spike and NS3 were not seen in previously reported lion SARS-CoV-2 sequences, ruling out the possibility that these are host-adapted mutations (*7*) (Appendix Figure 3). Further investigations could delineate whether changes in the spike protein, namely E156del, F157del, R158G, and K77T, are escape mutants or are associated with increased transmissibility or pathogenicity.

A nucleotide similarity comparison of the 4 lion SARS-CoV-2 sequences against the sequences available in GISAID and phylogenetic analysis revealed that the lion sequences closely matched with a representative human SARS-CoV-2 sequence of B.1.617.2 lineage, GISAID accession no. EPI_ISL_2463770, that comprises 152 viral genome pools collected from the same geographic region during the same month that the lions’ samples were collected (Figure; Appendix Figure 4). The park’s management strictly adhered to COVID-19 guidelines and did not introduce any new animals to the zoo during India’s widespread COVID-19 pandemic. The primary source of SARS-CoV-2 infection in the lions might have been an asymptomatic or paucisymptomatic person. Among the 9 infected lions, 7 were in the lion safari and shared a common habitat, shelter, feeding spaces, and water sources. The other 2 infected lions were on display in separate enclosures that shared a common moat. Because shared habitats offered opportunities for close physical contact, identifying genetically identical SARS-CoV-2 infections in these lions in a short period of time indicates the possibility of lion-to-lion transmission.

In conclusion, evidence of confirmed natural SARS-CoV-2 Delta variant infections in Asiatic lions in India justifies need for increased SARS-CoV-2 surveillance in wild animal species. In addition, strict biosecurity measures should be implemented for wild animals kept in captivity.

Appendix Additional methods and phylogenetic analyses of severe acute respiratory syndrome coronavirus 2 in Asiatic lions (*Panthera leo persica*), India.
