# Supplementary material for: SARS-CoV-2 Delta Variant among Asiatic Lions, India
Source: Emerg Infect Dis. 2021 Oct;27(10):2723–5. doi: 10.3201/eid2710.211500 (PMC8462327; doi:10.3201/eid2710.211500)
Supplement: Appendix — Additional methods and phylogenetic analyses of severe acute respiratory syndrome coronavirus 2 in Asiatic lions (Panthera leo persica), India. [file 21-1500-Techapp-s1.pdf]

Article DOI: https://doi.org/10.3201/eid2710.211500

SARS-CoV-2 Delta Variant among Asiatic Lions, India

Appendix

Supplementary Methods

SARS-CoV-2 Whole-Genome Sequencing on the Oxford Nanopore MinION platform

We performed whole-genome sequencing of severe acute respiratory syndrome coronavirus 2 (SARS-CoV-2) directly from nasal swabs of 4 Asiatic lions (*Panthera leo persica*) using the MinION (Oxford Nanopore Technologies, https://nanoporetech.com) sequencing platform. The lions tested included Jeya, a 3-year-old female; Shankar, an 18-year-old male; Niranjana, a 2-year-old female; and Pradeep, a 2-year-old male.

In brief, we performed tiling PCR spanning the whole genome of SARS-CoV-2 by using the Artic (https://artic.network) network primers. We performed cleaning and quantification of PCR products and used 100 ng of each sample to create a barcoded sequencing library by using the PCR Barcoding Kit (Oxford Nanopore). We used an Oxford Nanopore Technologies MinION with a R9.4.1 flow cell for sequencing, which yielded a total of 300 MB of data. To assemble the whole genome, we used guppy version 5.0.7 (Oxford Nanopore Technologies) for base calling and demultiplexing; and then we used Porechop (https://github.com/rrwick/Porechop) for adaptor removal. We mapped the readings to the SARS-CoV-2 reference genome (GenBank accession no. NC 045512) by using Minimap2 version 2.17 (r941) (*1*), and the called variations by using Nanopolish version 0.13.2 (https://github.com/jts/nanopolish) (*2*). After 2 rounds of Nanopolish, we generated 4 complete genomes.

References

1. Li H. Minimap2: pairwise alignment for nucleotide sequences. Bioinformatics. 2018;34:3094–100. PubMed https://doi.org/10.1093/bioinformatics/bty191

2. Loman NJ, Quick J, Simpson JT. A complete bacterial genome assembled de novo using only nanopore sequencing data. Nat Methods. 2015;12:733–5. PubMed https://doi.org/10.1038/nmeth.3444

Appendix Table 1. Characteristics and results of quantitative reverse transcription PCR on samples collected from 11 Asiatic lions, Arignar Anna Zoological Park, Chennai, India*

Lion identity	Age, y/sex	Sample type	C_t value			Results
			E gene	RdRp	N	
Jeya	3/F	Nasal swab	20.16	19.65	19.97	Positive
		Rectal swab	19.23	18.27	19.75	Positive
Padmnabhan	12/M	Nasal swab	18.90	18.10	18.50	Positive
		Rectal swab	27.41	26.75	27.79	Positive
Kavitha	18/F	Nasal swab	23.79	22.87	23.89	Positive
		Rectal swab	32.19	31.44	31.90	Positive
		Fecal sample	27.56	27.20	27.72	Positive
Shankar	18/M	Nasal swab	19.26	18.66	18.82	Positive
		Rectal swab	30.22	29.41	29.45	Positive
Neela	9/F	Nasal swab	22.71	23.22	22.57	Positive
		Rectal swab	25.09	24.74	24.46	Positive
		Fecal sample	–	–	–	Negative
Niranjana	2/F	Nasal Swab	19.68	19.04	19.17	Positive
		Rectal swab	38.61	38.14	33.69	Negative
		Fecal sample	–	–	–	Negative
Pradeep	2/M	Nasal swab	17.59	16.85	17.35	Positive
		Rectal swab	29.05	28.60	29.02	Positive
		Fecal sample	–	–	–	Negative
Vishnu	4/M	Fecal sample	24.34	23.80	24.72	Positive
Jeya@Bhuvana	12/F	Fecal sample	28.11	27.39	28.49	Positive
Veera	10/M	Fecal sample	–	–	–	Negative
Shiva	12/M	Nasal swab	–	–	–	Negative
		Throat swab	–	–	–	Negative
		Trachea sample	–	–	–	Negative
		Lymph node sample	–	–	–	Negative
		Lung sample	–	–	–	Negative

*C_t, cycle threshold; E, envelop gene; N, nucleocapsid; RdRP, RNA-dependant RNA polymerase; –, not detected.

Appendix Table 2. Amino acid substitutions and functional roles identified in different proteins encoded by severe acute respiratory syndrome coronavirus 2 detected in lions, India*

Amino acid substitutions	No. times reported (%)†	No. of countries‡	Mo. collected§	Accession no.¶	Functional roles of substitutions
M_I82T	51,777 (2.75)	99	Apr	hCoV-19/Indonesia/GO-NIHRD-PME20208/2020	–
N_D377Y	72,190 (3.83)	106	Feb	hCoV-19/USA/OH-ODH-SC1040172/2020	–
N_D63G	36,273 (1.93)	62	Apr	hCoV-19/Indonesia/GO-NIHRD-PME20208/2020	Antigenic drift; viral oligomerization interfaces
N_R203M	40,703 (2.16)	73	Mar	hCoV-19/Spain/NC-IBV-001370/2020	–
NS3_S26L	42,135 (2.24)	83	Mar	hCoV-19/USA/NY-NYCPHL-000016/2020	–
NS3_V88I	71 (0.00)	14	Mar	hCoV-19/Austria/CeMM0388/2020 hCoV-19/India/PB-ICMR-148040/2020 EPI_ISL_1165106 (B.1.36.8, Punjab, JUL-2020)	Viral oligomerization interfaces
NS7a_T120I	40,866 (2.17)	77	Feb	hCoV-19/Scotland/CVR01/2020	–
NS7a_V82A	39,371 (2.09)	66	Apr	hCoV-19/Indonesia/GO-NIHRD-PME20208/2020	Antigenic drift
NSP12_P323L	1,802,848 (95.76)	182	Oct	hCoV-19/Italy/MAR-UnivPM30_45476/2020	–
NSP15_K259R	4,692 (0.25)	54	Jul	hCoV-19/USA/CA-IGI-0320/2020	–
NSP2_P129L	13,166 (0.70)	91	Mar	hCoV-19/Japan/PG-1597/2020	–
NSP3_P822L	14,468 (0.77)	77	Feb	hCoV-19/USA/CA-CDPH018/2020	Host cell protein/RNA interaction; viral oligomerization interfaces
NSP4_D217N	2,516 (0.13	55	Mar	hCoV-19/England/BIRM-61F00/2020	–

Amino acid substitutions	No. times reported (%)†	No. of countries‡	Mo. collected§	Accession no.¶	Functional roles of substitutions
NSP4_F375S	1,136 (0.06)	33	Jun	hCoV-19/USA/WA-UW-10769/2020	–
NSP6_H11Q	3,639 (0.19)	48	Apr	hCoV-19/Italy/LOM-Pavia-41147/2020	–
Spike_D614G	1,839,357 (97.70)	185	Oct	hCoV-19/Italy/MAR-UnivPM30_45476/2020	Antigenic drift; virulence and host change; ligand binding; viral oligomerization interfaces
Spike_D950N	36,277 (1.93)	70	Mar	hCoV-19/Iran/K1r-108/2020	Viral oligomerization interfaces
Spike_E156G	32,810 (1.74)	62	Mar	hCoV-19/Panama/328688/2020	–
Spike_F157del	33,063 (1.76)	63	Jul	hCoV-19/USA/TX-HMH-MCoV-40913/2020	–
Spike_G142D	25,756 (1.37)	61	Mar	hCoV-19/England/BRIS-124CD4/2020	–
Spike_K77T	777 (0.04)	24	Dec	hCoV-19/Switzerland/ZH-ETHZ-431373/2020	–
Spike_L452R	101,257 (5.38)	108	Mar	hCoV-19/Denmark/ALAB-HH65/2020	Host and other changes; antigenic drift; antibody recognition sites
Spike_P681R	45,877 (2.44)	91	Apr	hCoV-19/Indonesia/GO-NIHRD-PME20208/2020	Increased rate of membrane fusion, internalization, and thus better transmissibility
Spike_R158del	33,071 (1.76)	63	Jul	hCoV-19/USA/TX-HMH-MCoV-40913/2020	Antibody recognition sites
Spike_T19R	320,114 (1.07)	64	Apr	hCoV-19/Indonesia/GO-NIHRD-PME20208/2020	Removes a potential N-glycosylation site that might also affect antigenic and other properties of this strain
Spike_T478K	56,250 (2.99)	79	Apr	hCoV-19/Indonesia/GO-NIHRD-PME20208/2020	Host and other changes; antigenic drift; host surface receptor binding; antibody recognition sites; viral oligomerization interfaces

*Reference sequence Wuhan-Hu-1 (GISAID accession no. EPI_ISL_402124) was used for comparison. –, no role identified.
†Percentage of sequences with this gene showing the same mutation.
‡Number of countries reporting the same mutations.
§Month first strain with this mutation was collected during 2020.
¶Accession number of the first strain to show the mutation.

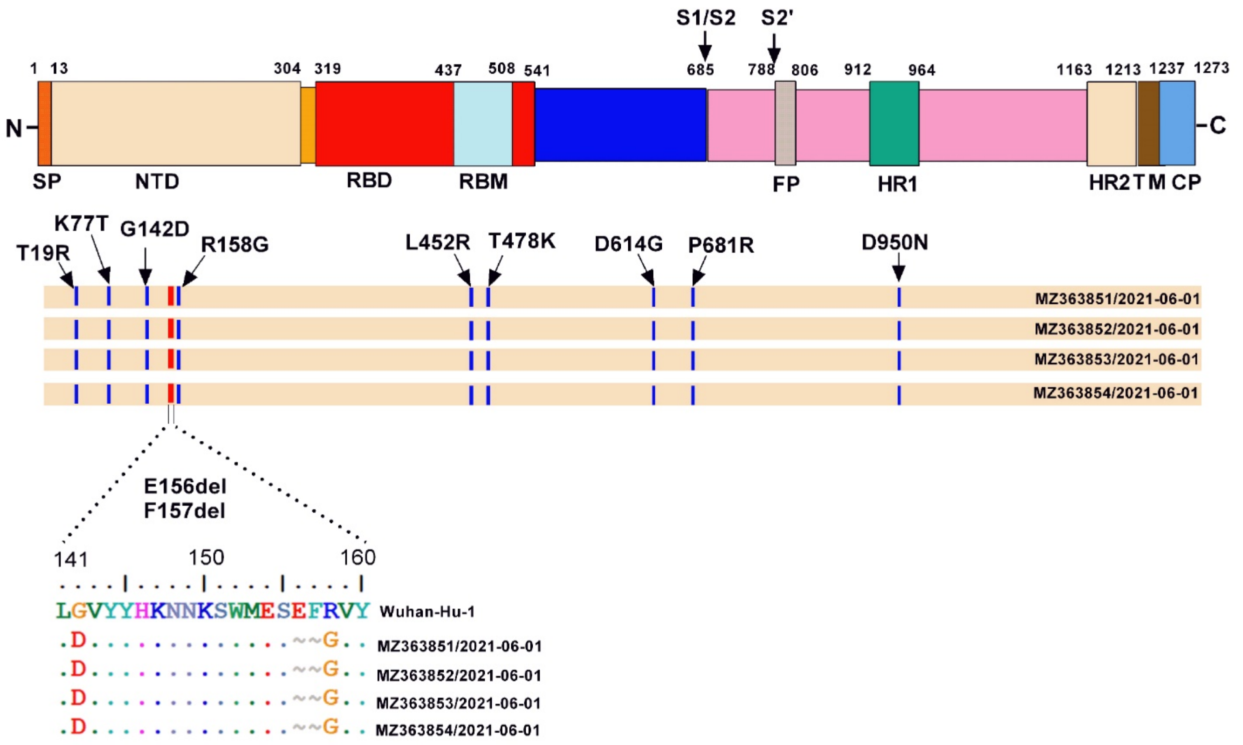

Appendix Figure 1. Comparison of amino acid changes detected in the spike protein (SP) of SARS-CoV-2 detected in Asiatic lions (*Panthera leo persica*), India. Wuhan-Hu-1 (GISAID accession no. EPI_ISL_402124) was used as the reference sequence. CT, cytoplasmic tail; FP, fusion peptide; HR, heptad repeat; NTD, N terminal domain; RBD, receptor binding domain; RBM, receptor binding motif; SARS-CoV-2, severe acute respiratory syndrome coronavirus 2; TM, transmembrane domain.

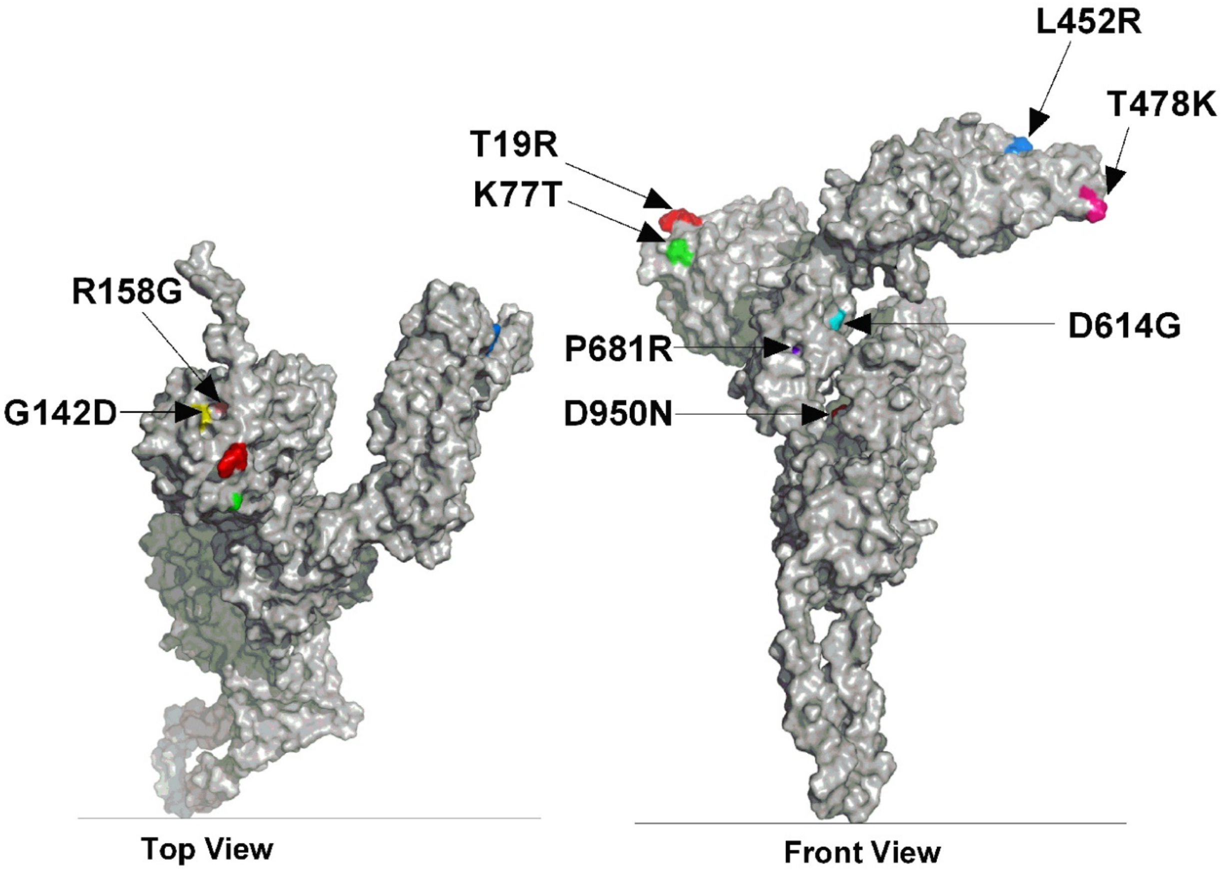

Appendix Figure 2. Mapping of amino acid substitutions noted on the structural model of the spike protein of SARS-CoV-2 detected in Asiatic lions (*Panthera leo persica*), India. Model represents spike protein of SARS-CoV-2 from Asiatic lion (GenBank accession no. MZ363851) from the top (left) and the front (right). Model was built by using I-TASSER (Yang Zhang Lab, https://zhanglab.ccmb.med.umich.edu/I-TASSER) and the PDB:6acc template. Red indicates T19R; green indicates K77T; and blue indicates L452R in both views; all other areas of interest are labeled with arrows. SARS-CoV-2, severe acute respiratory syndrome coronavirus 2.

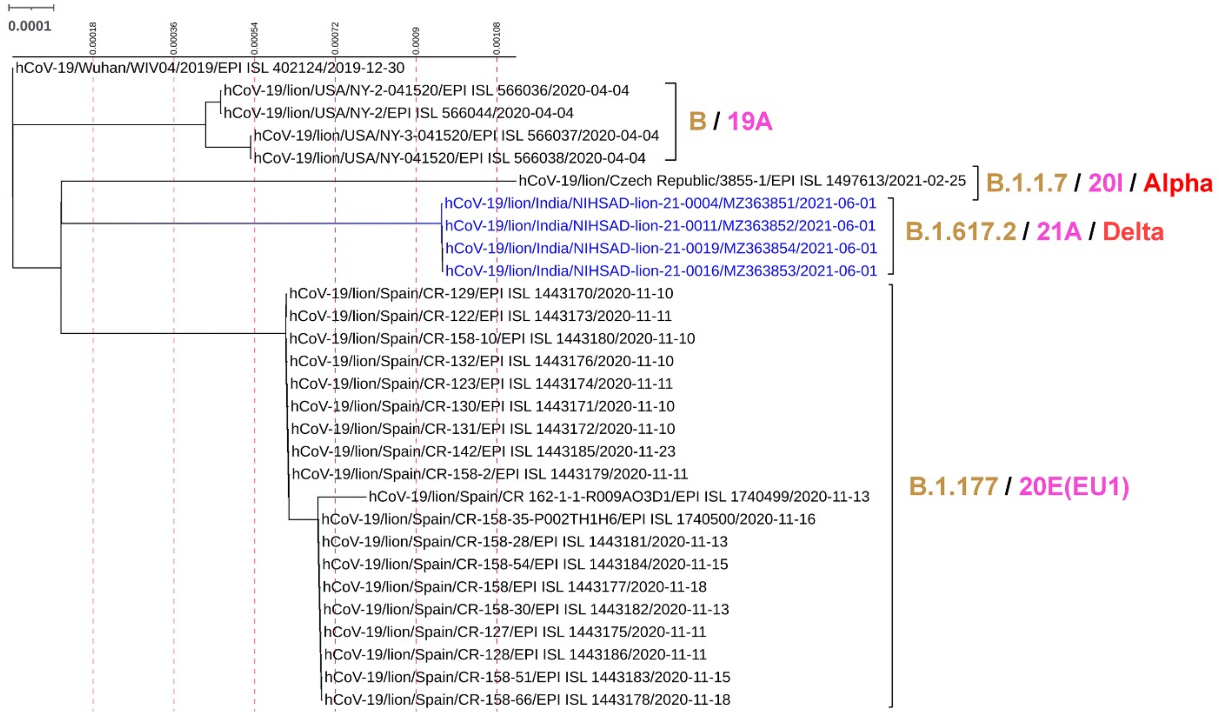

Appendix Figure 3. Maximum likelihood tree showing the phylogenetic relationship among SARS-CoV-2 detected in Asiatic lions (*Panthera leo persica*), India. Blue text indicates SARS-COV-2 sequences from this study. Comparison sequences were selected from available lion and tiger sequences in the GISAID. Scale bar indicates nucleotide substitutions per site. SARS-CoV-2, severe acute respiratory syndrome coronavirus 2.

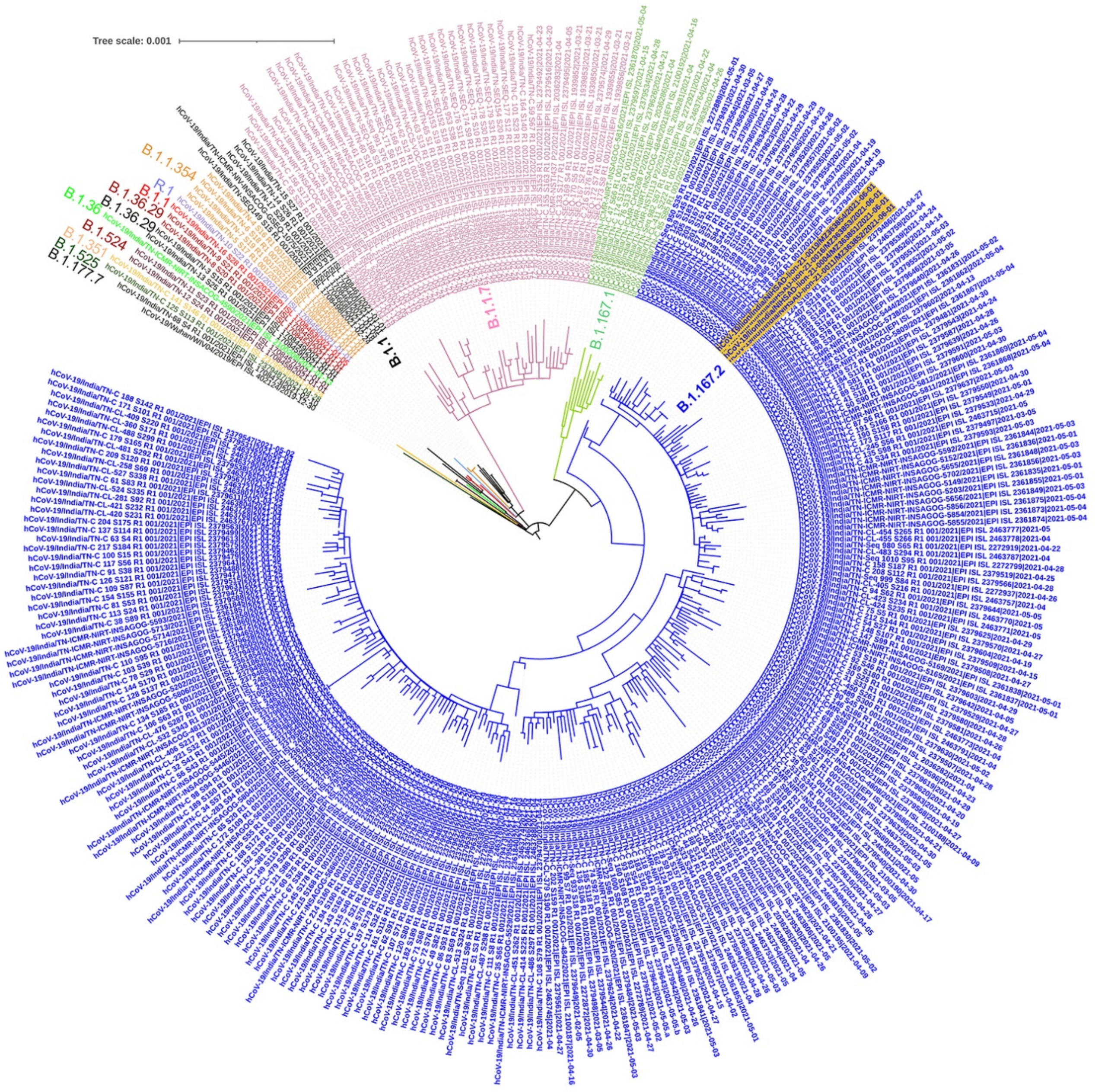

Appendix Figure 4. The phylogenetic analysis of SARS-CoV-2 detected in Asiatic lions (*Panthera leo persica*) and all available SARS-COV-2 sequences from Tamil Nadu, India. Colors represent different PANGO lineages; sequences in gold highlighting represent SARS-CoV-2 from lions in this study. Scale bar indicates nucleotide substitutions per site. SARS-CoV-2, severe acute respiratory syndrome coronavirus 2.
